# Social media for arthritis-related comparative effectiveness and safety research and the impact of direct-to-consumer advertising

**DOI:** 10.1186/s13075-017-1251-y

**Published:** 2017-03-07

**Authors:** Jeffrey R. Curtis, Lang Chen, Phillip Higginbotham, W. Benjamin Nowell, Ronit Gal-Levy, James Willig, Monika Safford, Joseph Coe, Kaitlin O’Hara, Roee Sa’adon

**Affiliations:** 1 The University of Alabama at Birmingham, FOT 802, 1720 2nd Ave S, Birmingham, AL 35294-3408 USA; 2 Global Healthy Living Foundation/CreakyJoints, Upper Nyack, NY USA; 3 Treato Ltd, Or Yehuda, Israel; 4 Weill-Cornell Department of Medicine, New York, NY USA; 5 Treato Ltd, Princeton, NY USA

**Keywords:** Rheumatoid arthritis, Social media, Twitter, Facebook, Tofacitinib, Tocilizumab, Etanercept, Herpes zoster

## Abstract

**Background:**

Social media may complement traditional data sources to answer comparative effectiveness/safety questions after medication licensure.

**Methods:**

The Treato platform was used to analyze all publicly available social media data including Facebook, blogs, and discussion boards for posts mentioning inflammatory arthritis (e.g. rheumatoid, psoriatic). Safety events were self-reported by patients and mapped to medical ontologies, resolving synonyms. Disease and symptom-related treatment indications were manually redacted. The units of analysis were unique terms in posts. Pre-specified conditions (e.g. herpes zoster (HZ)) were selected based upon safety signals from clinical trials and reported as pairwise odds ratios (ORs); drugs were compared with Fisher’s exact test. Empirically identified events were analyzed using disproportionality analysis and reported as relative reporting ratios (RRRs). The accuracy of a natural language processing (NLP) classifier to identify cases of shingles associated with arthritis medications was assessed.

**Results:**

As of October 2015, there were 785,656 arthritis-related posts. Posts were predominantly US posts (75%) from patient authors (87%) under 40 years of age (61%). For HZ posts (n = 1815), ORs were significantly increased with tofacitinib versus other rheumatoid arthritis therapies. ORs for mentions of perforated bowel (n = 13) were higher with tocilizumab versus other therapies. RRRs associated with tofacitinib were highest in conditions related to baldness and hair regrowth, infections and cancer. The NLP classifier had a positive predictive value of 91% to identify HZ. There was a threefold increase in posts following television direct-to-consumer advertisement (*p* = 0.04); posts expressing medication safety concerns were significantly more frequent than favorable posts.

**Conclusion:**

Social media is a challenging yet promising data source that may complement traditional approaches for comparative effectiveness research for new medications.

## Background

Social media is a burgeoning form of communication used by an increasingly large segment of the population. Social media is a vehicle for interactive communication where the producers of content (e.g. patients) can also be the consumers of content [1, 2]. By creating direct communication, social media can be seen as an equalizer, giving everyday people access to individuals and institutions with whom they would be unlikely to communicate otherwise. Social media permits the exchange of information and support to and between large numbers of people [3]. Despite its uptake by large segments of the population, the use of social media in healthcare has been less well-characterized. A variety of uses have been explored, ranging from simply conveying health information to messaging health promotion that is intended to motivate change in health behavior.

As one unique aspect of consumer-focused communication, the USA permits direct-to-consumer (DTC) advertising. These communications take a variety of forms including television, online communications, and print advertisements. DTC requirements include the need for “fair balance” that strikes the appropriate balance between risks of a particular medication and benefits for its approved indication, and these advertisements are under the governance of the U.S. Food and Drug Administration (FDA) [4]. However, there has been limited evaluation of how DTC messages are received by patients. It is possible that patients could be using social media to discuss and even report novel benefits or risks of newly licensed medications. This type of use would be particularly salient shortly after a product becomes available in the marketplace where real-world data are typically scant.

Given the limited information on the use of social media specific to arthritis and autoimmune conditions, the three main objectives of this analysis were to (1) descriptively characterize the demographics of people using social media to discuss rheumatoid arthritis (RA) and psoriatic arthritis (PsA) and psoriasis; (2) to evaluate the suitability of social media as a data source for drug safety research, particularly for the study of recently licensed molecules, and (3) classify the content and timing of the posts that these social media users are contributing, with a particular focus on communication related to newer biologic drugs and small molecules in relation to DTC advertising launch dates.

## Methods

### Data source

We used the Treato [5] technology to analyze all publicly-available social media data including Facebook, blogs, and discussion boards for posts mentioning inflammatory arthritis (e.g. rheumatoid arthritis or psoriatic arthritis) and specific mentions (by name) of four medications used for RA (e.g. tofacitinib, tocilizumab, abatacept and etanercept). These represented the three newest medications approved for RA (tofacitinib, tocilizumab and abatacept) and an anti-TNF agent (etanercept) with greater specificity for arthritis in that it is not used to treat inflammatory bowel disease.

The Treato platform identifies and collects publicly available user-generated content on health topics from over 10,000 sources. These sources include the publicly available data on social networks such as Facebook and Twitter, discussions forums and blogs. Over 2.5 billion posts were analyzed from these sources. Natural language processing (NLP) algorithms analyze this content to identify medical concepts mentioned in text and extract patients’ self-reported descriptions of their health conditions and medications. More specifically, Treato takes medical terms and maps them to formal concepts in a medical ontology [6]. This process includes resolving conceptual synonyms of medical terms (e.g., myocardial infarction and heart attack); resolution of patient-specific terms (e.g., “pain in my joints” and “my joints hurt”) to medical terms; word-sense disambiguation algorithms (e.g., “BP” could refer to bi-polar disorder, blood pressure, or a bisphosphonate medication); and medication synonyms (e.g., generic and brand names for the same medication). Following the textual processing, posts are tagged with concepts, rather than just keywords, which allows for finer search and filtering capabilities. For example, a search for the herpes zoster concept would find posts referring to either “herpes zoster” or “shingles” (synonym), but not to posts referring to “shingles vaccine” (unless these posts also refer to “shingles” separately). In addition, the data are analyzed to extract patients’ specific experiences, making a distinction between posts that merely mention a disease and posts in which patients report having the disease. Other examples include identifying patients switching from drug A to drug B, and drug side-effect reports. Individual posts can be manually reviewed and classified by a subject matter expert.

The unit of analysis was an individual post and each term in the post, as described below. Although these data are dynamic and change every minute, the data extracted and used for this analysis extended through October 2015. The characteristics of individuals contributing posts of interest were described in relation to demographics and geography, as consistent with the privacy settings of the various social media sources that permit such disclosure and their availability in the data.

For posts mentioning medications of interest, the Treato platform was used to create word clouds to summarize the frequencies of co-occurrence mentions, with the size of the text represented as the relative frequency of each term compared with other terms. The data were subsequently analyzed using disproportionality methods (see subsequent text) to evaluate the content of what was being discussed and to evaluate whether there were novel topics associated with RA drugs of interest.

### Analysis of pre-specified and empirically identified health-related events and concerns associated with specific RA medications

For the analysis of medication safety, we pre-specified two known health conditions and associated drugs recognized in large phase III clinical trials in RA (herpes zoster and tofacitinib, and gastrointestinal perforation and tocilizumab). Disease and symptom-related treatment indications (e.g. arthritis, joint pain) were manually redacted and excluded from the database. The co-occurrence frequencies of each medication and either of the two events (mentioned in the same post) were compared between RA medications and reported as pairwise odds ratios (ORs), and were analyzed using Fisher’s exact test. For this analysis, the threshold for statistical significance was *p* < 0.01.

Additionally, in a third analysis, empirically identified mentions of safety events and symptoms with at least five events that were co-occurring with mentions of RA drugs were analyzed using disproportionality analysis, one of the common methods used to analyze FDA Adverse Event Reporting System data [7, 8]. Results were characterized first as relative reporting ratios (RRRs, probability for event associated with a drug exposure divided by the probability for the event in the whole population) and as proportional reporting ratios (PRRs), which could be conceptually interpreted as the probability of an event in drug exposure A divided by the probability of an event in drug exposure B.

### Analysis of Twitter data related to arthritis medication safety and tolerability

The top social media influencers were identified using Symplur, which maintains a controlled database in which each healthcare hashtag is vetted, checked for level of critical mass, and grouped appropriately with other relevant hashtags and Twitter accounts. According to Symplur’s Healthcare Hashtag project that aggregates and categorizes Twitter healthcare data, top “influencers” were identified based upon the Twitter accounts that tweet out the most content, and the accounts with content that is the most retweeted [9].

All drug-specific posts (“tweets”) for the four medications of interest were extracted from Twitter from 2014 to 2015, identified using drug names anywhere in the post (and not only drug-specific hashtags), as was a 20% random sample of tweets related to rheumatoid or psoriatic arthritis, regardless of their mention of specific medications. These were reviewed manually to determine whether they were related to arthritis medications of interest and/or contained content associated with DTC advertising. Tweets were classified manually into the a-priori specific categories of a potential safety event, a safety concern (i.e. fear about a future event), or medication tolerability (e.g. injection site burning or pain). Representative examples of tweets within each category were selected for review to illustrate additional medical themes and were shown as direct quotes, redacting information that might identify a contributor’s identity.

DTC launch dates of new USA television advertisements for etanercept, tofacitinib, and abatacept were obtained from the manufacturers of these agents. We compared the volume of tweets in the 2 weeks following the DTC launch to the 2 weeks prior to the DTC launch date, using the paired *t* test to test the hypothesis that there was an increased volume of social media posts following each new DTC advertising launch date. This analysis was conducted in both the tweets specifically mentioning DTC advertisement content and again reanalyzed, using all available Twitter data.

### Performance of automated classification via natural language processing compared to manual review for drug-associated herpes zoster events

Treato contains an NLP-based classifier to identify medical events and conditions to improve specificity beyond simple text mining. To understand the performance characteristics of this classifier operating within Treato, the system was queried for posts mentioning both herpes zoster and one of the four drugs of interest (etanercept, abatacept, tocilizumab or tofacitinib). This pre-filtering included matching posts that mentioned one of the four drugs (or their synonyms), and contained a reference to herpes zoster (or one of its synonyms).

This initial pre-filtering yielded 602 posts, of which a random sample of 425 were selected for manual review, to determine whether they represented a true case of patient-reported herpes zoster or not. A true case would be a patient who reported experiencing shingles (herpes zoster), and a non-true case would reflect that even though the drug and shingles (herpes zoster) were mentioned in the post, the author was not specifically claiming he or she had experienced shingles. This association did not require the author or the manual reviewer to assert or infer causality of the herpes zoster event to be related to the drug. These posts were evaluated by the Treato NLP classifier as to whether they represented a bona-fide case of drug-associated shingles and were compared to the gold standard of manual review. A contingency table was generated that reported sensitivity (recall), specificity, positive predictive value (precision) (PPV), and negative predictive value (NPV) compared to the gold standard of manually reviewed cases. All analyses were performed by SAS version 9.4 (Cary, NC, USA). Patient consent was not required for this study given that all data sources were publically available. The university institutional review board governed the study protocol.

## Results

**Fig. 1:**
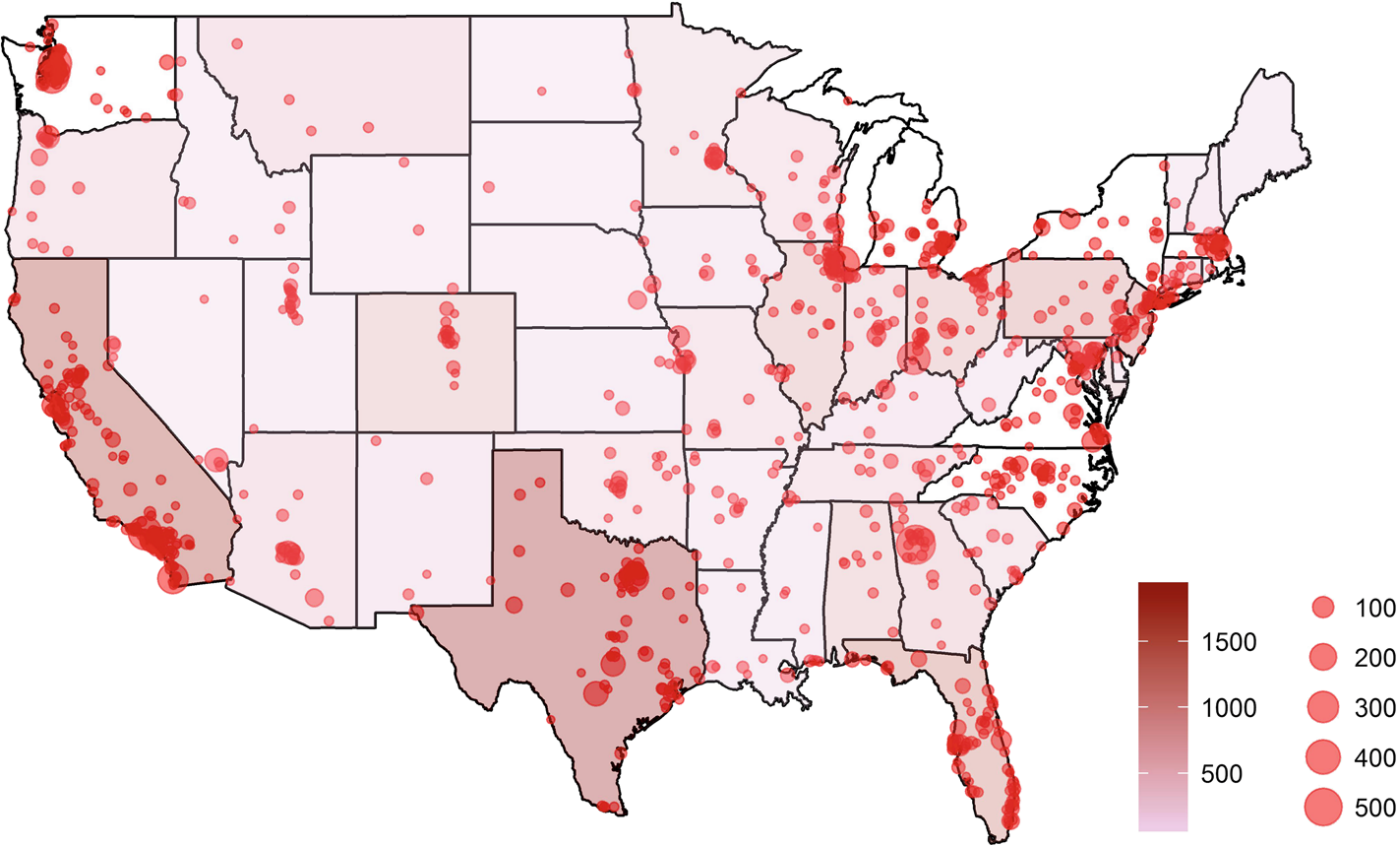
USA locations associated with geolocatable* posts mentioning “rheumatoid arthritis”. *Red dots indicate cities where posts originated (when available); when not available at city-level precision, *shading* indicates the density of posts at a state level

As of October 2015, there were 785,656 arthritis-related posts discussing tofacitinib (n = 1279), etanercept (n = 23,530), abatacept (n = 4424), and tocilizumab (n = 1791). Posts were predominantly from the USA (75%) and originating from the geographic distribution shown in Fig. 1, predominantly from patients as authors (87%). The age distribution of individuals contributing the posts was <19 (18%), 19–29 (23%), 30–39 (20%), 40–49 (17%), 50–54 (17%), and 65+ (6%) years. Facebook (and all of its groups) was the most common platform for social media discussions. Based upon data from Symplur (accessed 26 May 2016), the most common sources for social media mentions of RA on Twitter included a mix of professional patient advocacy organizations and individual patients. The top USA-based influencers in relation to RA were @CreakyJoints, @ACRheum, and @RheumaArthritis.

In the Treato platform, analysis of posts mentioning herpes zoster (n = 1815) associated with tofacitinib were compared with other RA medications and are shown in Table 1 (upper half). Many ORs were significantly increased for tofacitinib compared to other RA therapies. Similarly, many of the associations between posts mentioning gastrointestinal perforation and tocilizumab (n = 13) were significant compared to other RA therapies (Table 1, lower half).

**Table 1:** Pairwise odds ratios for posts mentioning herpes zoster associated with tofacitinib and gastrointestinal perforation associated with tocilizumab versus other medications used for rheumatoid arthritis

Medication exposures	Number, by drug	Odds ratio (95% CI)
Herpes zoster^a^		
Tofacitinib vs. infliximab	22 vs. 405	2.45 (1.59–3.77)
Tofacitinib vs. golimumab	22 vs. 23	2.34 (1.30–4.21)
Tofacitinib vs. rituximab	22 vs. 120	2.03 (1.29–3.20)
Tofacitinib vs. adalimumab	22 vs. 613	1.82 (1.19–2.79)
Tofacitinib vs. etanercept	22 vs. 446	1.67 (1.09-2.57)
Gastrointestinal perforation^b^		
Tocilizumab vs. rituximab	13 vs 7	12.62 (5.03 – 31.64)
Tocilizumab vs. adalimumab	13 vs. 59	6.85 (3.76 – 12.51)
Tocilizumab vs. infliximab	13 vs 60	5.99 (3.29 – 10.92)
Tocilizumab vs. abatacept	13 vs. 6	5.23 (1.99 – 13.79)
Tocilizumab vs. certolizumab	13 vs. 10	3.47 (1.52 – 7.93)

^a^Non-significant associations for other rheumatoid arthritis (RA) medications are not shown. ^b^Non-significant associations for other RA medications are not shown, nor where estimates were potentially unstable based upon cell counts <5 in at least one exposure group (contrasts with etanercept and golimumab were removed for this reason)

**Fig. 2:**
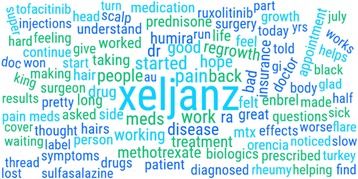
Example word cloud, describing associations of terms in discussions of tofacitinib (Xeljanz). *Size of the text* reflects the frequency of concept mentions in the Treato sample

As an example, the co-occurrence frequencies of concepts associated with tofacitinib were shown as a word cloud (Fig. 2). Co-therapies commonly given with each of these drugs were frequently mentioned (e.g. prednisone, methotrexate). Terms that were somewhat more specific for tofacitinib compared to other RA therapies included “head” and “hair”. Based on a disproportionality analysis, the health conditions uniquely mentioned in posts on tofacitinib and tocilizumab were summarized and ranked in descending order by proportional reporting ratios. Consistent with the word cloud, for tofacitinib they were highest for alopecia *universalis*, alopecia *totalis*, male pattern baldness, alopecia *areata*, vitiligo, and skin pigmentation; all these PRRs were significant at *p* < 0.0001: for tocilizumab, they were systemic sclerosis, cytokine release syndrome, giant cell arteritis, endocarditis, sore tongue, and high cholesterol.

Extending the analysis beyond only examining term co-occurrence frequencies, the performance characteristics of the automatic NLP classifier within Treato were examined with respect to its ability to identify bona-fide HZ events reported by patients that were associated with drugs of interest. In Table 2, and compared to manual review of posts as the gold standard, the PPV (precision) of the automated NLP classifier was 91%, sensitivity (recall) was 71%, and specificity was 84%. When the sensitivity of the algorithm was increased to 100% (i.e. classifying every occurrence as a report), the PPV was lower but still high (71%), and specificity was reduced to 0% (not shown).

**Table 2:** Performance characteristics of natural language processing classifier to identify patient-reported herpes zoster events associated with arthritis medications of interest

	Manual review of all posts mentioning shingles			
Automated Treato NLP algorithm	Post contained shingles report	Post did not contain shingles report	Total	
Shingles case detected in post	213	20	233	PPV (precision) = 91% (87–95%)
Shingles case was not detected in post	89	103	192	NPV = 54% (46–61%)
Total	302	123	425	
	Sensitivity (recall) = 71% (65–76%)	Specificity = 84% (76–90%)		

Performance characteristics are reported as proportion (95% confidence interval). *NLP* natural language processing, *PPV* positive predictive value, *NPV* negative predictive value

Focusing specifically on Twitter data, 10,023 tweets were manually reviewed. These mentioned arthritis drugs (n = 8032), specifically etanercept (n = 4486, 44.8%), tofacitinib (n = 1637, 16.3%), tocilizumab (n = 1021, 10.2%) and abatacept (n = 888, 8.9%), plus tweets mentioning no medication but that were derived from the 20% random sample of posts related to inflammatory arthritis (n = 1991, 19.9%). Of these, 481 (4.8%) were related to DTC advertisements, the majority of which were specifically related to television advertisements (n = 290); for most of the remainder, the type of media that the DTC advertisement appeared in could not be inferred.

Of the 8032 tweets mentioning the four drugs of interest, 46 (<1%) identified a potential safety event, 277 (3.4%) mentioned a tolerability problem, and 327 (4.1%) listed a safety concern. A number of medical themes were present in the social media posts that could be at times medically related. Table 3 shows representative examples of such themes. As described, patient posts were focused on issues such as access to medications, safety concerns, expectations of efficacy, and overall satisfaction with treatment. Themes were similar between arthritis medications. Among the 481 related to DTC advertisements, 59 (12.3%) mentioned concerns about drug safety. The most frequent safety concerns specifically mentioned, in descending order of frequency, were cancer, death, fatal infections and gastrointestinal perforation. Fewer than 5% were positive with respective to describing favorable aspects of the medications.

**Table 3:** Examples of health-related content themes for social media posts mentioning anti-TNF biologic agents or other RA medications

Patient quote	Health-related theme
I have been waiting two days for my enbrel and now I have wait another two days for the "order to process“. I could get crack faster!	Access, potential low adherence
30 second push for #enbrel because it burns like battery acid. I have this down to an art and that ain't right	Tolerability, potential low adherence
The pharmaceutical drug #Orencia has too many dangerous side effects to be advertised on TV!	Safety concerns
I'm sick of #Humira & #Enbrel commercials for moderate to severe #psoriasis! This (XXX) ain't workin for me! Enbrel only covers my joints.	Dissatisfaction, desire to change therapy
I don't get why they (my skin lesions) keep popping up. I took my enbrel. That's supposed to halt everything, but it's not working.	Unrealistic expectations of efficacy
The day enbrel puts whatever the hell is in these shots in a pill will probably one of the best days of my life!	Dissatisfaction with formulation, possible interest in oral medication (e.g. janus kinase inhibitor)

There were six product-specific DTC advertising campaigns for etanercept, abatacept and tofacitinib in 2014–2015. Using all the Twitter data without restriction to tweets mentioning DTC ads, there was no significant association between the timing of new DTC ad launches and the volume of social media posts for each of the advertised RA medications. However, after restricting analysis to the data mentioning DTC advertisements (n = 481), tweets mentioning DTC advertising were disproportionately approximately threefold greater in the 2 weeks following the DTC launch than in the two weeks prior to it (*p* = 0.04).

## Discussion

Using a software platform that searched all public-facing social media, we examined content related to inflammatory arthritis (e.g. RA, psoriatic arthritis) and the medications used for its treatment. We found a large volume of posts related to this health condition and associated drug therapy. Using social media content, we confirmed known associations between mentions of herpes zoster and tofacitinib, and gastrointestinal perforation and tocilizumab, as expected based on large phase III clinical trials. We also found social media content related to hair regrowth and skin re-pigmentation, a novel observation seen with patients receiving tofacitinib. Finally, we observed a small but measurable impact of DTC advertising on posts from patients commenting on these advertisements. However, fewer than 5% of such communications were positive, and many invoked medication-related safety fears expressed by patients.

This analysis examined social media in relation to arthritis and its treatments, and we note that social media has been studied for a variety of health conditions including cancer [10, 11], diabetes [12, 13] and obesity [14]. Our interest was focused on the application of social media to medical product safety and patient-perceived safety, a topic that has been recently examined across a number of therapeutic areas [15–18]. Prior work has identified correlation between adverse events as recorded in electronic health records and patient concerns assessed in social media data [19]. In another study, content analysis of social media posts related to the recall of an over-the-counter medication found that the use of Facebook allowed patients to interact with the manufacturer in relation to communicating product recall messaging and related risk, and to promote patient support and self-efficacy [20].

While social media content has been commonly used to convey medication-related information, much less work has been done to understand whether the information is both understood clearly and made actionable, nor to assess what patients are saying about the content that they encounter. In an analysis of Facebook pages related to the 20 most-searched health conditions on Google, the largest percentage of pages (32.2%, n = 168) were dedicated to marketing or promotion; 20.7% (n = 108) to information; and 13.0% (n = 68) to social support [21]. In 2008, the drug industry spent $4 billion on DTC advertising, of which less than 4% was directed toward online media [22]. However, expenditure on DTC advertising has increased substantially over time, and across all forms of communication, DTC spending grew to US$5.4 billion in 2015 [23]. Overall, the pharmaceutical industry spent an estimated US$1.93 billion on digital advertising in 2016 [24].

Because the world of social media is largely unmonitored, it has the potential to be co-opted or thwarted, whether intentionally or unintentionally. For example, the Movember Foundation sought to raise awareness of health risks including prostate and testicular cancer, but only 0.7% of all tweets occurring during the campaign were focused on this topic, and discussions in the USA were dominated by the discussions around moustache grooming [25]. More generally, social media used to support messaging from pharmaceutical, for-profit healthcare companies, or individual stakeholders, can carry similar risks. For example, online pharmacies of questionable trustworthiness may market to patients; non-experts may share information of questionable validity; and consumer perceptions of the benefits or risks of a treatment may become exaggerated [26]. Encouragingly, research conducted on DTC advertising [27] has found some success in communicating medication information to lay audiences [28–30]. In this project that examined patient sentiment captured via social media related to DTC advertising, we found a small but measurable impact on the volume of comments related to advertising of medications after the launch of television-based DTC advertisements for biologic therapies. However, patients’ sentiments about the medications were typically to be unfavorable, more often expressing concerns about safety and tolerability problems than expressing hope that the medication might provide an effective treatment option.

In an empiric example of using social media data to identify drug-associated medical events, we found that the PPV (precision) of an automated NLP classifier to find bona-fide cases of HZ reported by patients was high (91%), sensitivity was reasonable (71%) and specificity was also high (84%). One might conclude that for a large-scale project where manual review is not feasible, the system might be used in an automated fashion with an acceptably high PPV to detect early safety signals. However, if time and resources permit, manual review of social media posts is helpful to identify more true cases and improve the specificity of classifying safety events.

The generalizability and reach of social media deserves some mention. According to Pew’s Demographics of Social Media study that polled 1907 participants by phone in 2015, 1612 of whom were Internet users, 72% of adult Internet users use Facebook and 23% of all Internet users use Twitter [31]. The vast majority of younger adults used Facebook, including 82% of adults ages 18–29 years and 79% of adults ages 30–49 years. Social media is also growing in popularity among older adults; among adults with Internet access, nearly two thirds of middle-aged adults and about half of individuals over 65 years of age use Facebook. Facebook is more popular among women than men, and 77% of women are users [31]. Twitter is more popular among urban residents than it is among their suburban or rural counterparts and there is a larger age gap among users; 30% of online adults under 50 years of age use Twitter, compared with 11% of online adults ages 50 years and older [31]. Perhaps contrary to expectation, the so-called “digital divide” is not necessarily present among social media users. People of color who have Internet access have been shown to be more likely to use social media than their white counterparts [32]. Moreover, there is some evidence that social media may give traction to health interventions with “hard-to-reach” populations, such as Spanish-speaking Latino men who have sex with men (MSM) by using different platforms including Facebook, Craigslist and smartphone applications [33].

The strengths of our study include a novel look at a global information source that has been infrequently studied in healthcare, using a robust platform that extracted medical terms, resolved synonyms, and enabled quantitative analysis. However, there are some limitations inherent to such data. The demographics of people using social media are still evolving, but social media is disproportionately used by younger people. In our sample, 60% of patients were younger than age 40 years. Additionally, the availability of demographic information was predicated on people being willing to use relaxed privacy settings on their own characteristics, including geolocation. Comfort with sharing this information is likely to be variable by patient community and is likely to change over time. Additionally, while we analyzed individual posts, tweets, and terms in them, future analyses that can extract terms not only within posts but across posts, within the same thread or even across threads, are likely to be useful, given that patients may be constrained in the length of their posts and tweets. For example, Twitter limits tweets to 140 characters, although this may change over time [34]. Similarly, longitudinal analysis of an author’s posts across a timeline could open the door to additional types of analysis. Finally, manual review of raw posts can be accommodated within the Treato platform, but was labor-intensive, given the sometimes limited relevance of Twitter data to our use case. However, the pre-filtering capability of the Treato platform could make this type of manual review process much more efficient.

## Conclusions

Social media is a challenging, yet promising data source that may complement traditional methods to study new medications shortly after licensure. While social media data may be useful for hypothesis generation for safety evaluation or even off-label indications, results should be interpreted with caution, and should assist in guiding focused follow-up studies.

## Abbreviations

DTC: Direct To Consumer
FDA: U.S. Food and Drug Administration
HZ: Herpes zoster
NLP: Natural language processing
ORs: Odds ratios
PPV: Positive predictive value (precision)
PRRs: Proportional reporting ratios
PSA: Psoriatic arthritis
RA: Rheumatoid arthritis
RRRs: Relative reporting ratios
TNF: tumor necrosis factor
